# P-836. Non-Beta Lactam Agents for Definitive Treatment of Ampicillin-Susceptible *Enterococcus* Bacteremia: A Single Center Experience

**DOI:** 10.1093/ofid/ofae631.1028

**Published:** 2025-01-29

**Authors:** HeeEun Kang, Asif khan, Justin J Kim, Isabella W Martin, Richard A Zuckerman

**Affiliations:** Loyola University Medical Center; Dartmouth Hitchcock Medical Center, Lebanon, New Hampshire; RUSH University Medical Center, Chicago, Illinois; Dartmouth-Hitchcock Medical Center, Lebanon, NH; Dartmouth-Hitchcock Medical Center, Lebanon, NH

## Abstract

**Background:**

*Enterococcus* bacteremia (EB) is a serious infection with high morbidity and mortality. While anti-enterococcal beta-lactams (BL) remain the mainstay of therapy against ampicillin-susceptible (AS) EB, non-beta-lactam (NBL) antimicrobial agents are also used to treat ASEB. Few studies evaluate NBL use for definitive therapy of ASEB.

**Figure:**
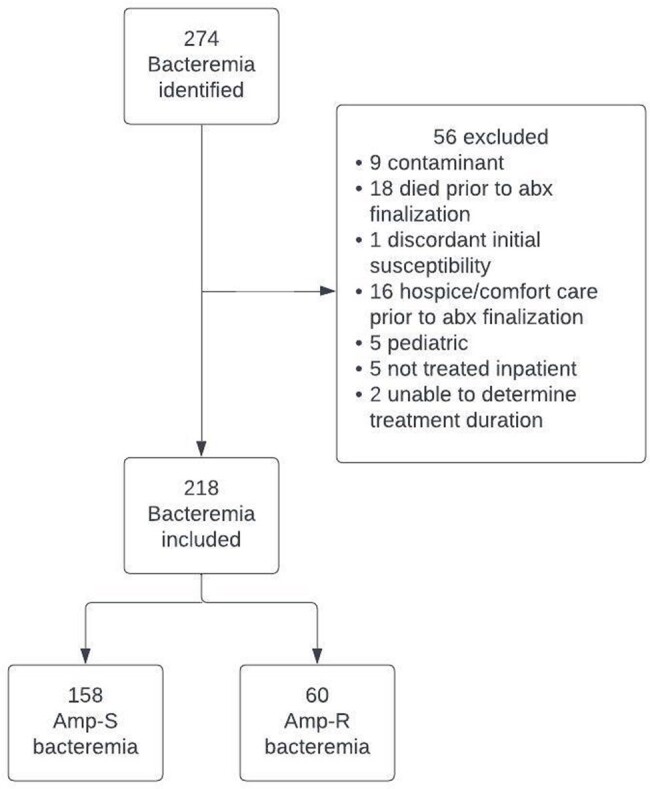

CONSORT flow diagram of patients included in study.

**Methods:**

A single-center retrospective study identified all ASEB episodes between 1/1/2016-12/31/2021 (Figure 1). Patient, microbiological, infection, treatment and outcomes were compared between those who received NBL versus BL for ASEB. Multivariable logistic regression analysis was used to determine factors associated with NBL use.

**Figure:**
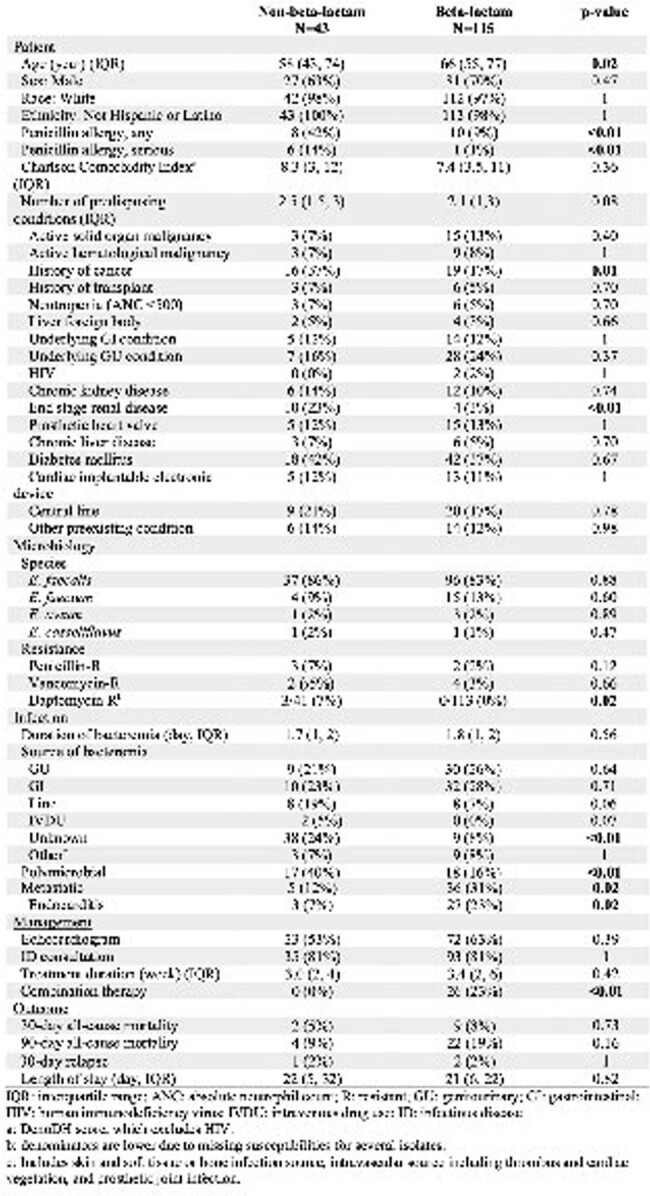

Baseline characteristics of patients treated with non-beta-lactam vs beta-lactam for definitive therapy for ampicillin-susceptible Enterococcus bacteremia.

**Results:**

158 episodes of ASEB in 153 patients were included (Table 1). 43 episodes (27%) were treated with NBL for definitive therapy. Factors associated with NBL therapy were younger age (58 vs 66 years old, p=0.02), reported history of any penicillin allergy (42% vs 9%, p< 0.01) and serious penicillin allergy (14% vs 1%, p< 0.01), history of cancer (37% vs 17%, p=0.01), end-stage renal disease (ESRD; 23% vs 3%, p< 0.01), unknown source of bacteremia (24% vs 8%, p< 0.01), polymicrobial bacteremia (40% vs 16%, p< 0.01), lack of metastatic foci of infection (12% vs 31%, p=0.02), and lack of endocarditis (7 vs 23%, p=0.02). Combination therapy was used in 23% of those treated with BL therapy compared to no patients receiving NBL therapy (p< 0.01).

For NBL vs BL therapy, there was no difference between all-cause 30-day mortality, all-cause 90-day mortality, or 30-day relapse rate.

In a multivariable logistic regression model (Table 2), NBL therapy was more likely in those with: younger age (AOR 0.95, p=0.001), any penicillin allergy (AOR 5.76, p=0.001), history of cancer (AOR 5.23, p=0.001), ESRD (AOR 11.70, p=0), and polymicrobial bacteremia (AOR 4.20, p=0.001).

**Figure:**
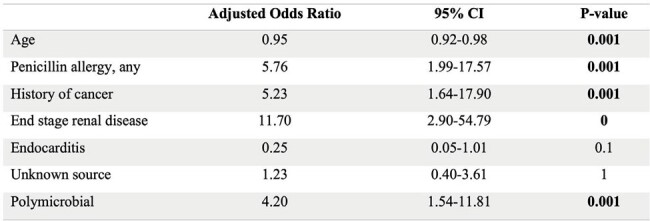

Factors associated with use of non-beta-lactam antibiotic for definitive therapy of ampicillin-susceptible Enterococcus bacteremia: multivariable analysis.

**Conclusion:**

NBL was used as definitive treatment in 27% of ASEB. Penicillin allergy, access and dosing for patients on hemodialysis, younger host, and polymicrobial infection are factors associated with NBL use. This real-life experience suggests NBL can be successfully used to treat non-severe ASEB. Further studies are needed to determine the efficacy and stewardship implications of NBL use for ASEB.

**Disclosures:**

**All Authors**: No reported disclosures

